# Identification and validation of circulating miRNAs as potential new biomarkers for severe liver disease in patients with leptospirosis

**DOI:** 10.1371/journal.pone.0257805

**Published:** 2021-09-27

**Authors:** Natthaya Chuaypen, Umaporn Limothai, Pattapon Kunadirek, Pornchai Kaewsapsak, Patipark Kueanjinda, Nattachai Srisawat, Pisit Tangkijvanich

**Affiliations:** 1 Center of Excellence in Hepatitis and Liver Cancer, Department of Biochemistry, Faculty of Medicine, Chulalongkorn University, Bangkok, Thailand; 2 Excellence Center for Critical Care Nephrology, Faculty of Medicine, Chulalongkorn University, Bangkok, Thailand; 3 Department of Biochemistry, Faculty of Medicine, Chulalongkorn University, Bangkok, Thailand; 4 Department of Microbiology, Faculty of Medicine, Chulalongkorn University, Bangkok, Thailand; Kunming University of Science and Technology, CHINA

## Abstract

**Background:**

Leptospirosis, a global zoonotic infectious disease, has various clinical manifestations ranging from mild self-limiting illness to life-threatening with multi-organ damage, including liver involvement. This study was aimed at identifying circulating microRNAs (miRNAs) as novel biomarkers for predicting severe liver involvement in patients with leptospirosis.

**Methods:**

In a discovery set, 12 serum samples of patients with anicteric and icteric leptospirosis at initial clinical presentation were used for miRNA profiling by a NanoString nCounter miRNA assay. In a validated cohort, top candidate miRNAs were selected and further tested by qRT-PCR in serum samples of 81 and 16 individuals with anicteric and icteric leptospirosis, respectively.

**Results:**

The discovery set identified 38 significantly differential expression miRNAs between the two groups. Among these, miR-601 and miR-630 were selected as the top two candidates significantly up-regulated expressed in the icteric group. The enriched KEGG pathway showed that these miRNAs were mainly involved in immune responses and inflammation. In the validated cohort, miR-601 and miR-630 levels were significantly higher in the icteric group compared with the anicteric group. Additionally, these two miRNAs displayed good predictors of subsequent acute liver failure with a high sensitivity of 100%. On regression analysis, elevated miR-601 and miR-630 expression were also predictive of multi-organ failures and poor overall survival.

**Conclusion:**

Our data indicated that miRNA expression profiles were significantly differentiated between the icteric and anicteric groups. Serum miR-601 and miR-630 at presentation could potentially serve as promising biomarkers for predicting subsequent acute liver failure and overall survival in patients with leptospirosis.

## Introduction

Leptospirosis, a global zoonotic infectious disease caused by *Leptospira interrogans*, is prevalent especially in developing countries and tropical areas [1]. In Thailand, the incidence of leptospirosis is 5–10 per 100,000 population, with the mortality rate of approximately 1% [2]. The clinical manifestation of leptospirosis varies in its severity from a mild, self-limited febrile illness to a fulminant life-threatening course involving multiple organ systems, particularly the liver, kidneys, brain and lungs [1, 3]. In the context of liver involvement, patients with leptospirosis are commonly found to have mild to moderate abnormal liver function tests including elevations of direct bilirubin and transaminases levels. Moreover, approximately 5–10% of infected patients could develop severe icteric form, which is associated with a high mortality rate of 5–15% [4, 5]. Additionally, extensive hepatocyte necrosis leading to acute liver failure has been reported, which emphazises the significance of this rapid and potentially irreversible complication [3]. Despite its important impact, predictive markers of developing severe hepatic manifestation of leptospirosis are not yet available in routine practice. Therefore, identifying reliable serum biomarkers for early prognostic prediction of leptospirosis with severe liver involvement are greatly needed and would be of clinical benefit.

Recently, microRNAs (miRNAs), a class of small non-coding endogenous RNAs of 18–24 nucleotides, has been shown to regulate gene expression by degrading or suppressing the translation of target mRNAs [6]. In addition, miRNAs participate in several biological activities including homeostasis, immune activation and control of infection. Accordingly, detecting aberrant expression of miRNAs in human biofluids could reflect various pathophysiological conditions, including infectious process [7]. To date, circulating miRNAs have been identified as potential circulating biomarkers for diagnosis and prognosis of various infectious diseases including malaria, tuberculosis, human immunodeficiency virus and viral hepatitis [8]. Nonetheless, studies of circulating miRNAs in patients with leptospirosis are currently very limited.

In this study, we compared global miRNA expression profiling in serum specimens obtained from icteric and anicteric patients with leptospirosis by NanoString nCounter platform. Additionally, we selected candidate miRNAs for further analysis by quantitative real-time PCR (qRT-PCR) in another set of serum specimens to evaluate their association with disease severity and overall survival of patients with leptospirosis. Finally, the roles and functions of the candidate miRNAs were also predicted by the analysis of functional enrichment of their target genes.

## Materials and methods

### Study design and patients

In this study, patients with confirmed leptospirosis, who were attended at 15 hospitals in Sisaket province, Thailand between December 1, 2017 to November 30, 2018 were included. The diagnostic criteria for leptospirosis were confirmed by one of the following methods: (1) 16S rRNA gene partial sequencing to identify leptospiral isolates (2) *Leptospira* agglutination examined by dark-field microscopic agglutination test (MAT) with positive results defined as single titer ≥400 or four-fold rising titers between acute and convalescent phases (3) specific primers for pathogenic *Leptospira* DNA detecting by PCR. Serum samples were collected at initial clinical presentation and then stored at -80°C until further analysis.

The patients were devided into 2 groups namely anicteric and icteric leptospirosis according to serum total bilirubin levels at a cut-off value of <3 and ≥3 mg/dL, respectively. Patients with suspected unconjugated hyperbilirubinemia (e.g. hemolysis) were excluded if direct bilirubin levels were < 20% of the total bilirubin levels [9]. All patients had given their written informed consent, and the study was performed following the Helsinki Declaration and Good Clinical Practice guidelines. The study protocol was approved by the ethical committee, Faculty of Medicine, Chulalongkorn University, and the ethical committee for research in the human subject of Si Sa Ket Hospital (COA No.004 REC No. 071/2560).

### RNA extraction from serum samples

Serum samples (200 μl) were used for total RNA extraction using the miRNeasy Serum/Plasma Kit (Qiagen, Gaithersburg, MD, USA) according to the manufacturer’s instruction. *C*. *elegans* synthetic miR-39 mimic were spiked into each serum samples as an internal control. The RNA concentration and purity were measured using the DeNovix DS-11 Spectrophotometer (DeNovix Inc, Wilmington, DE).

### NanoString nCounter System Assays

A total of 12 serum samples (9 and 3 samples from the anicteric and icteric groups, respectively) were randomly selected from the stored samples to investigate the expression profile of 800 human miRNAs using the nCounter® Human v3 miRNA miRNA Expression Assays (NanoString Technologies, Seattle, USA). Three microliters of 100 ng of total RNA was processed according to the manufacturer’s protocol. All counts were collected and captured by the nCounter Digital Analyzer with 280 fields of view per sample. Raw count data were managed in nSolver^TM^ software (v4.0; NanoString). For each miRNA count data was subtracted with background subtraction using the geometric mean of the negative controls. Profiling data were then normalized by the average (geometric mean) of the positive controls, and the geometric mean of the top 100 most highly-expressed microRNAs. Differential miRNAs expression between groups were defined as absolute log_2_ fold-change ≥3.0 and false discovery rate (FDR) with adjusted *P*-values ≤0.05. The raw datasets from nCounter miRNA Expression Assay were deposited at the NCBI Gene Expression Omnibus (GEO) (http://www.ncbi.nlm.nih.gov/geo/) under accession number GSE179426.

### miRNAs validation by qRT-PCR

Top candidate miRNAs obtained from the expression profiles were selected for qRT-PCR validation. The selection of candidate miRNAs based on 2 criteria (1) the consistency of up- or down-regulated expressions (log_2_ fold-change >3.0, *P* <0.05) in serum samples of icteric patients and (2) known to be involved in immune response and inflammation. Following these criteria, only up-regulated miR-601 and miR-630 were selected for further qRT-PCR validation.

Total RNA was polyadenylated with synthesis stem-loop-poly A(SLpolyA; GTCGTATCCAGTGCAGGGTCCGAGGTATTCGCACTGGATACGACAAAAAAAAAAAAAAAAAAVN). After polyadenylated, reverse transcription to cDNA was performed by using RevertAid First Strand cDNA Synthesis Kit (Cat No. 1622, Thermo Scientific, USA). The miRNA levels were quatified from cDNA in duplicate by using SYBR Green (QPCR Green Master Mix HRox, Cat No. BR0500402, Biotechrabbit, Germany) and real-time PCR (StepOnePlus Real-time PCR System, Applied Biosystems, USA) as previously described [10]. The primers for two miRNAs were designed as the following: miR-601: 5’-TGGTCTAGGATTGTTGGAGGAG-3’ (forward), miR-630:5’-AGTATTCTGTAC CAGGGAAGGTAA-3’(forward), and SL-polyA-R: 5’-GCAGGGTCCGAGGTATTCG-3’ (universal reverse). Additionally, miR-16-5p (forward, 5’-CAGCACGTAAATATTGGCG-3’), representing one of the most stably expressed miRNAs in serum samples based on several studies [11–14], was chosen as an appropriate endogenous reference for the normalization of miRNAs expression. Finally, the relative expression in comparison to control of the individual miRNA was performed.

### Gene ontology and KEGG pathway enrichment analysis

Potential targeted genes of candidate miRNAs were predicted by using miRNA target prediction programs (TargetScan) [15]. Then, clusterProfiler R package [16] was used to analyze the involvement of target genes in biological processes and functional analysis [17]. Enriched Gene Ontology (GO) term was identified by the cut-off of FDR adjusted *P*<0.05. The Kyoto Encyclopedia of Genes and Genomes (KEGG) pathways were used to predict the target genes of two selected miRNAs with FDR adjusted *P*-value cut off < 0.01.

### Protein–protein interaction (PPI), network construction and analysis

The protein-protein interaction (PPI) networks of common gene targets between these two miRNAs was constructed by STRING database [18]. The interaction of gene network was further visualized by the Cytoscape (version 3.7.1) with assigning source node, target node and combined score ≥ 0.9 as edge attribute [19]. The mRNA-miRNA network was constructed according to nodes with degree of >6 and visualized the regulation network using the Cytoscape 3.7.1 software [19].

### Statistical analysis

All statistical analyses were calculated with SPSS Version 22 (SPSS, Chicago, IL) and GraphPad Prism 8 (GraphPad Software Inc., California, USA). The NanoString analysis was performed using nSolver Analysis Software (Version 4.0). Continuous variables were expressed as means ± standard deviation (SD), median and interquartile range (IQR). Student’s t test or Mann-Whitney test was performed to analyze the differences between two continuous variables. Categorical variables were displayed as numbers with percentages and were analyzed by the Chi-square test. The correlations between miRNAs and clinical parameters were performed using Spearman rank test. The association of miR-601and miR-630 with acute liver failure was calculated using individual univariate binary logistic regression analyses. The log transformation of miR-601and miR-630 values was applied to construct parametric data (Kolmogorov > 0.05). Odds ratios (OR) and 95% confidence intervals (CI) were then calculated. In addition, the area under receiver-operating-characteristic (AUROC) was performed to assess the diagnostic performance of candidate miRNAs. *P*-values below 0.05 were considered statistically significant.

## Results

### Patient characteristics

Overall, 97 patients with confirmed leptospirosis were included in this study. Among these, 81 and 16 individuals were classified into the anicteric and icteric groups, respectively (Table 1). Compared with the anicteric group, patients with icteric leptospirosis had significantly lower platelet count, glomerular filtration rate (GFR), and serum albumin levels. Moreover, icteric patients had higher serum creatinine, total bilirubin, direct bilirubin, SGOT levels, modified sequential organ failure assessment (SOFA) score, mortality rate and length of hospital stay, as well as higher rates of mechanical ventilation and ICU admission. Other baseline characteristics were comparable between groups.

**Table 1:** Demographic data of the studied cohorts.

Clinical Characteristics	Anicteric (N = 81)	Icteric (N = 16)	*P*-value
Age; years	51.8±17.2	47.3±14.0	0.337
Sex; male	65 (80.3)	16 (100.0)	0.052
BMI; kg/m^2^	22.0±3.8	21.8±2.5	0.839
Alcoholism	5 (6.2)	1 (6.3)	1.000
Smoking	38 (46.9)	6 (37.5)	0.928
Day of fever	3.0 (1.0–4.0)	3.5 (2.3–5.0)	0.056
Body temperature; °C	38.2±1.2	38.3±1.6	0.755
Systolic blood pressure; mmHg	113.3±22.9	102.8±25.9	0.107
Mean arterial pressure; mmHg	80.0 (73.3–87.8)	75.0 (65.2–82.4)	0.805
Hemoglobin; g/dL	12.1±1.9	11.7±7.1	0.805
WBC; cellsx10^3^μL	10.4±4.3	10.2±6.4	0.895
Platelets; cellsx10^3^μL	151.8±123.0	46.8±32.4	<0.001*
Creatinine; mg/dL	1.1 (0.9–1.4)	2.5 (1.2–5.9)	<0.001*
Glomerular filtration rate; mL/min	70.5±31.1	42.2±34.1	<0.001*
HCO3-; mmol/L	24.1±4.3	22.2±7.3	0.346
Total bilirubin; g/dL	1.1 (0.7–1.7)	5.0 (3.4–9.6)	<0.001*
Direct bilirubin; g/dL	0.6 (0.3–0.9)	3.7 (2.2–6.8)	<0.001*
SGOT; U/L	52.0 (34.3–94.5)	105.0 (50.0–159.0)	0.016*
SGPT; U/L	36.5 (22.0–77.3)	56.0 (37.3–95.0)	0.068
Albumin; g/dL	3.6±0.7	2.8±0.7	<0.001*
Leptospiremia (F. qPCR LipL32)	385.0 (106.7–2585.6)	996.0 (128.8–3039.8)	0.383
Positive hemoculture	6 (7.4)	1 (6.3)	0.978
SOFA score	2.0 (1.0–5.0)	8.0 (5.0–10.0)	<0.001*
Death	3 (3.7)	3 (18.8)	0.022*
Length of hospital stay; days	4.0 (2.3–6.0)	7.0 (3.3–12.5)	0.029*
Mechanical ventilation	6 (7.4)	5 (31.3)	0.006*
ICU admission	4 (4.9)	5 (31.3)	<0.001*

Data are expressed as mean ± SD, median (IQR) or n (%) as appropriate

*P-values<0.05.

### miRNAs expression profile in serum samples

**Fig 1:**
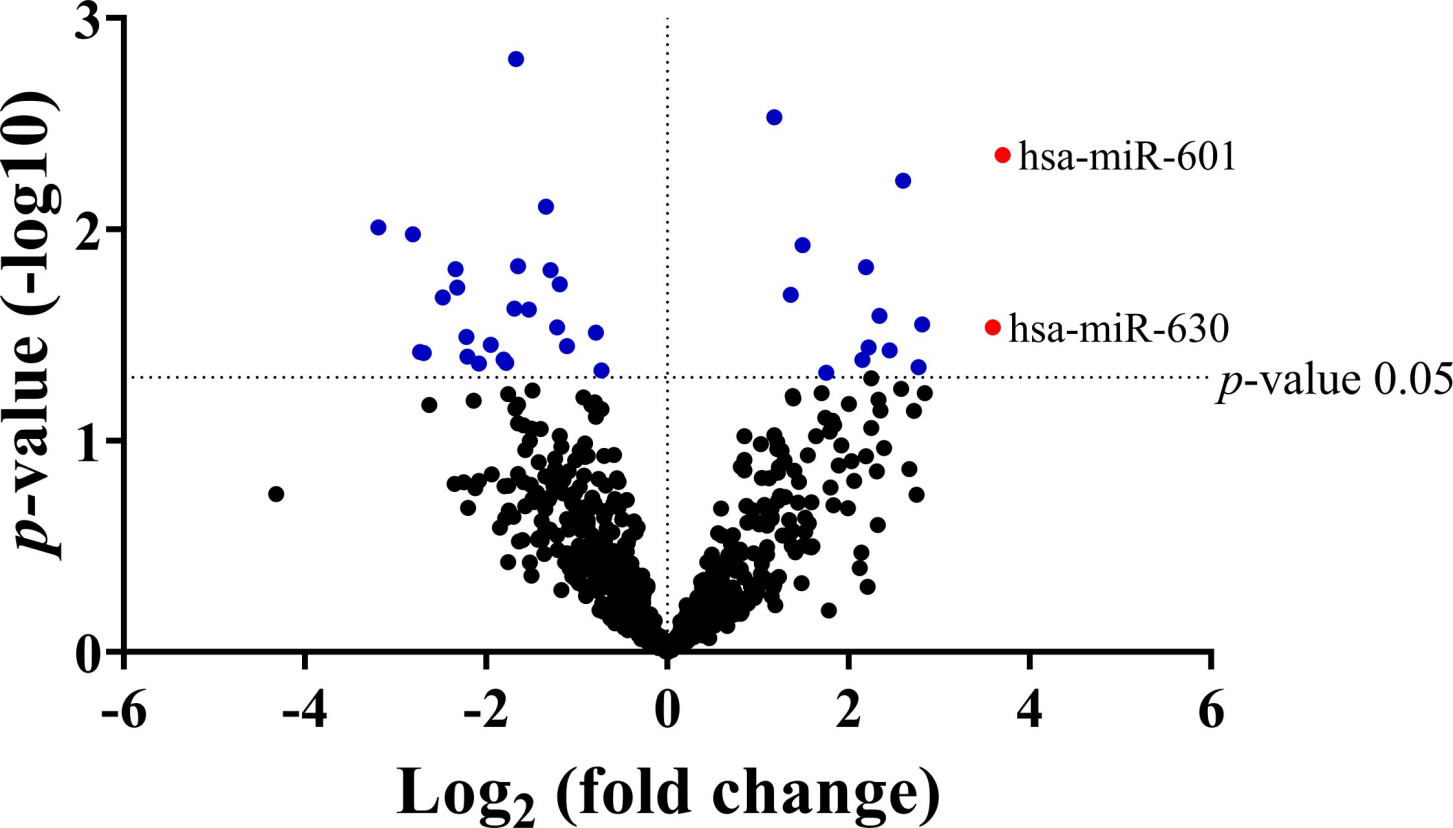
Volcano plot of miRNAs expression profiling. The blue dots represent miRNAs that demonstrate a significant change in expression (left side; downregulated miRNAs, right side; upregulated miRNAs). The red dots showed the two candidate miR-601 and miR-630. The significance value was indicated with a *p*-value <0.05.

As the ratio of anicteric:icteric was approximately 5:1, we decided to apply similar proportion of samples in a discovery set. Accordingly, serum samples of 9 anicteric patients and 3 icteric patients were selected to identify candidate miRNAs. Of 800 miRNAs determined by the NanoString Platform, 38 miRNAs had significant differential expression between the two groups, as demonstrated in S1 Table and S1 Fig. Among these, only two up-regulated miR-601 and miR-630 had a fold change higher than 3.0 (Fig 1). Therefore, we selected these two miRNAs for further qRT-PCR validation.

### Validation of miRNA ratios by RT-qPCR

**Fig 2:**
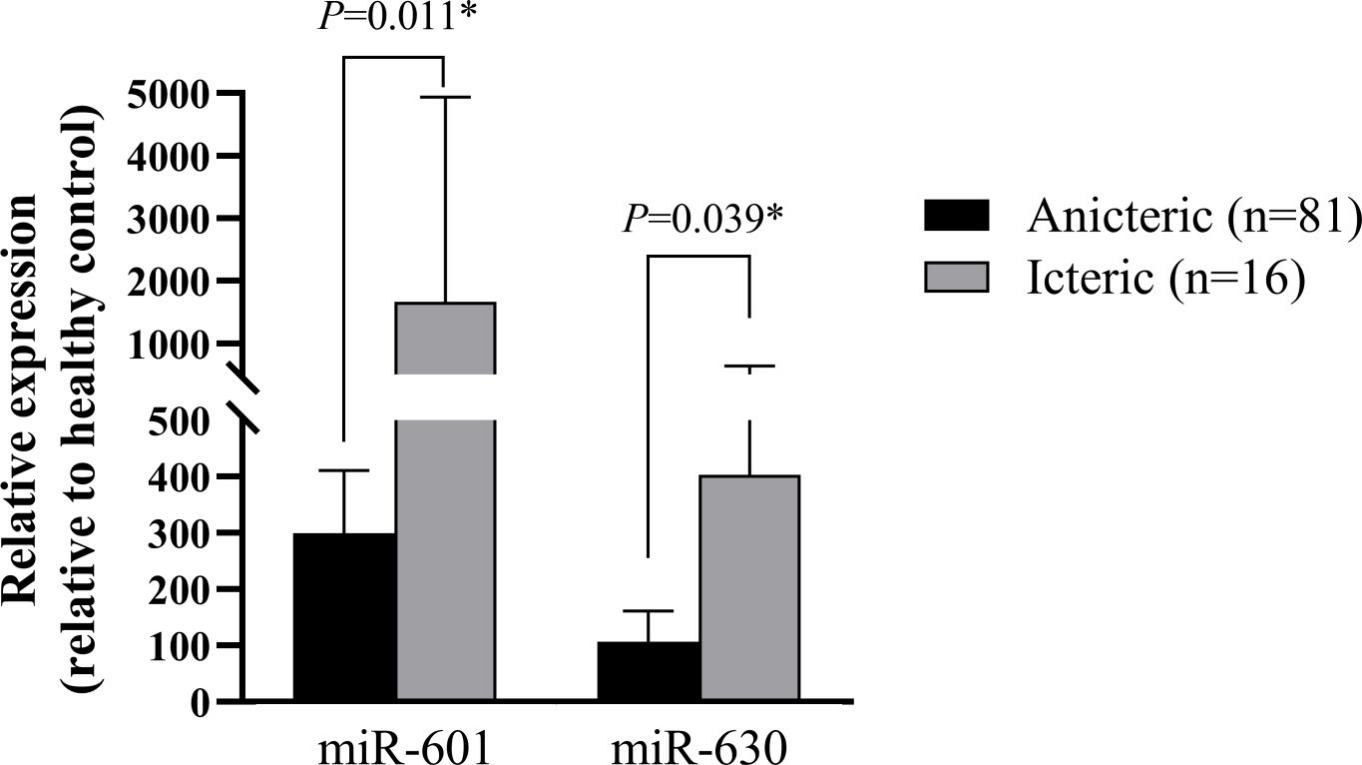
Relative expression of serum miR-601 and miR-630 in patients with the anicteric and icteric groups.

The validated set consisted of all samples of patients with leptospirosis in this cohort (81 anicteric and 16 anicteric). In this respect, up-regulated miR-601 and miR-630 identified in the discovery set were further investigated in both groups of patients. Relative expression miRNAs levels of the subjects in each group are plotted in Fig 2. The results indicated that circulating miR-601 level was significantly higher in the icteric group than the anicteric group (1,666.2±3,272.4 vs. 300.0±1,000.3, *P =* 0.011). Similarly, serum miR-630 level was significantly higher in the icteric group compared with the anicteric group (403.5±940.8 vs. 107.1±488.0, *P =* 0.039).

### Relationship between serum miRNAs levels and clinical parameters

In this study, the correlation between serum miRNAs levels and clinical parameters at baseline was examined. Serum miR-601 had a negative correlation with hemoglobin (r = -0.215, *P* = 0.034), hematocrit (r = -0.210, *P* = 0.039), lymphocyte (r = -0.261, *P* = 0.010), glomerular filtration rate (r = -0.301, *P* = 0.003), serum albumin level (r = -0.332, *P* = 0.001), total protein (r = -0.277, *P* = 0.009) and sodium levels (r = -0.261, *P* = 0.011). Contrastly, serum miR-601 had a positive correlation with blood urea nitrogen (r = 0.359, *P* = 0.001), serum creatinine level (r = .307, *P* = 0.002), total bilirubin (r = 0.337, *P* = 0.001), direct bilirubin (r = 0.289, *P* = 0.004) and alkaline phosphatase (r = 0.227, *P* = 0.025)

For miR-630 had a negative correlation with lymphocyte (r = -0.254, *P* = 0.012), glomerular filtration rate (r = -0.292, *P* = 0.004), serum albumin level (r = -0.294, *P* = 0.004), total protein (r = -0.235, *P* = 0.027) and serum chloride level (r = -0.209, *P* = 0.044). In contrast, serum miR-630 had a positive correlation with blood urea nitrogen (r = 0.299, *P* = 0.004), serum creatinine level (r = 0.310, *P* = 0.002), total bilirubin (r = 0.258, *P* = 0.011) and direct bilirubin (r = 0.251, *P* = 0.014).

### Serum miRNAs as biomarkers of hepatic dysfunction in leptospirosis

**Fig 3:**
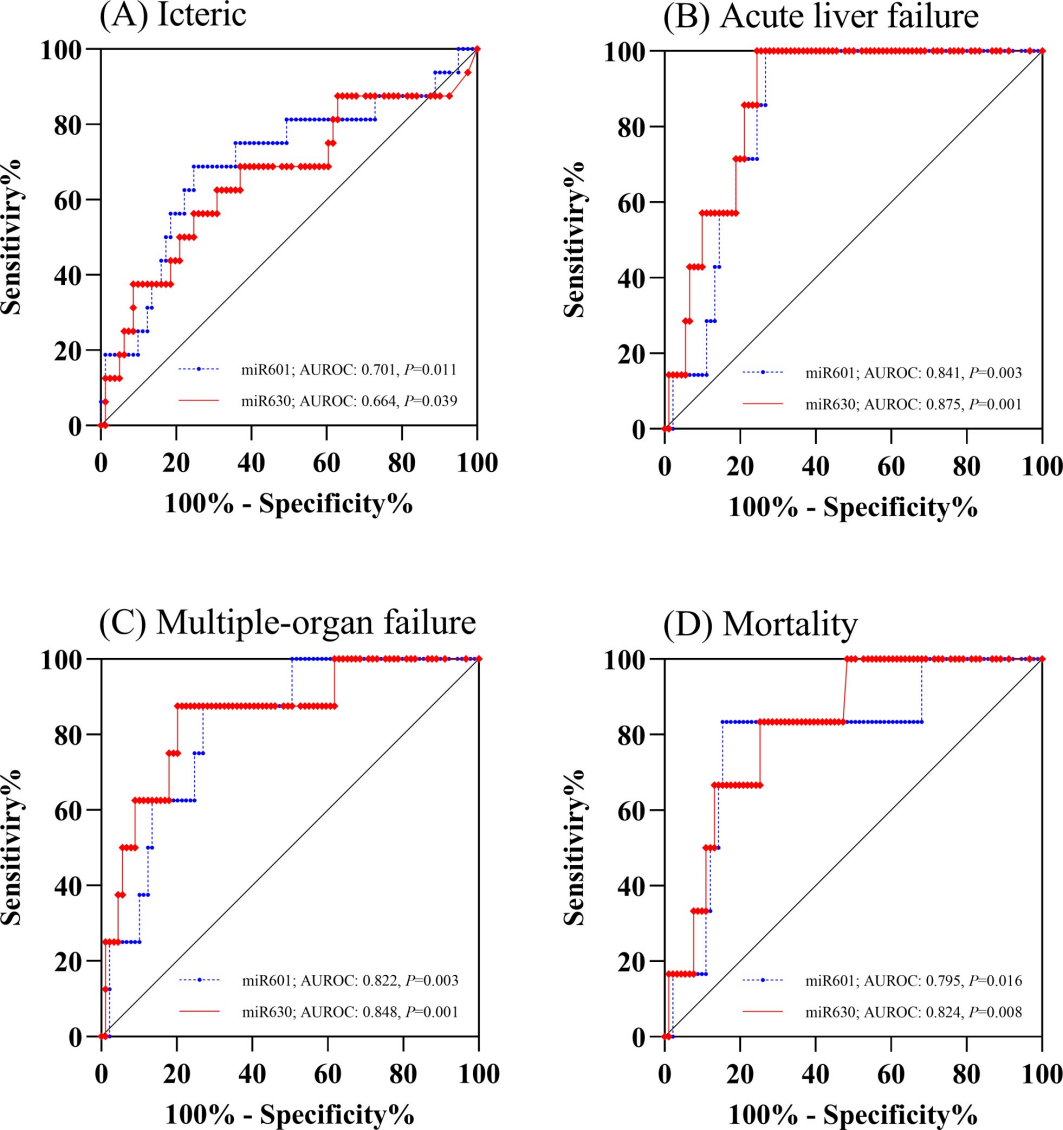
ROC curve analysis of serum miR-601 and miR-630 for icteric status (A), acute liver failure (B), multiple organ failure (C), and mortality (D).

The ROC curves for miR-601 and miR-630 were generated to calculate their diagnostic accuracies. As shown in Fig 3A, serum miR-601 levels could discriminate between the icteric and anicteric groups with an AUROC of 0.70 (95%CI;0.55–0.86, *P* = 0.011). Likewise, serum miR-630 levels could discriminate between the icteric and anicteric groups with an AUC of 0.66 (95%CI;0.50–0.82, *P* = 0.039). Based on the ROC, the optimal cut‐off values for miR-601 and miR-630 levels in detecting icteric leptospirosis were 112.4 and 2.9, respectively. At this cut-off level, its sensitivity and specificity were approximately 69% and 75% for miR-601 and approximately 69% and 63% for miR-630 (Table 2).

**Table 2:** The sensitivity and specificity of serum miR-601 and miR-630 in the diagnosis of icteric leptospirosis, acute liver failure, multiple organ failure and mortality.

Biomarker	Cut-off values	Sensitivity (%)	Specificity (%)
**Icteric leptospirosis**			
miR601	112.4	69%	75%
miR630	2.9	69%	63%
**Acute liver failure**			
miR601	112.4	100%	73%
miR630	6.8	100%	76%
**Multiple organ failure**			
miR601	112.4	88%	73%
miR630	10.7	88%	80%
**Mortality**			
miR601	235.8	83%	85%
miR630	7.9	83%	75%

The prognostic value of miR-601 and miR-630 expression to predict acute liver failure was further evaluated. As shown in Fig 3B, the AUROCs for miR-601 and miR-630 are 0.84 (95%CI;0.76–0.93, *P* = 0.003) and 0.87 (95%CI;0.79–0.96, *P* = 0.001), respectively. Based on the ROC, the optimal cut‐off values for miR-601 and miR-630 levels were 112.4 and 6.8, respectively. At this cut-off point, the sensitivity and specificity was approximately 100% and 73% for miR-601 and approximately 100% and 66% for miR-630 (Table 2). We also evaluated both miRNAs in predicting multiple organ failure. The results showed that the AUROC of miR-601 and miR-630 with regard to multiple organ failure were 0.82 (95%CI;0.70–0.94, *P* = 0.003) and 0.85 (95%CI;0.71–0.99, *P* = 0.001), respectively (Fig 3C). The AUROC curves of a combined use of miR-601 and miR-630 were also analyzed (S2 Fig). However, the combination of both miRNA expression did not improve the predictive performance.

Serum miR-601 and miR-630 levels were entered into the univariate analysis together with other variables that might influence the prognosis of acute liver failure. These baseline factors included age, gender, BMI, white blood cells, platelets, serum bicarbonate, SGOT, SGPT, alkaline phosphatase, leptospiremia, miR-601, and miR-630. The data revealed that prognostic factors of acute liver failure were platelets, miR-601, and miR-630 (Table 3).

**Table 3:** Variables associated with acute liver failure in patients with leptospirosis.

Variables	Catagory	Univariate			
		OR	(95%CI)		*P*-value
Age (years)	≥60 vs. <60	1.29	0.23	7.06	0.767
Gender	Female vs. Male	-	-	-	-
BMI (kg/m^2^)	≥24.9 vs. <24.9	1.56	0.17	14.17	0.694
White blood cells (cellsx10^3^μL)	≥10,000 vs. <10,000	1.17	0.25	5.51	0.846
Platelets (cellsx103μL)	<75,000 vs. ≥75,000	14.54	1.6	126.79	0.015*
Bicarbonate (mmol/L)	<25 vs. ≥25	7.23	0.84	62.65	0.073
SGOT (U/L)	≥40 vs. <40	3.37	0.38	29.23	0.271
SGPT (U/L)	≥40 vs. <40	2.67	0.49	14.52	0.254
Alkaline phosphatase	≥112 vs. <112	1.03	0.22	4.86	0.974
Leptospiremia (F. qPCR LipL32)	≥400 vs. <400	2.56	0.47	13.88	0.277
Log_10_ miR-601	≥235.8 vs. <235.8	6.67	1.35	32.90	0.020*
Log_10_ miR-630	≥7.9 vs. <7.9	18.55	2.12	162.57	0.008*

OR, odd ratio; CI, confident interval

*P-value<0.05.

### Serum miRNAs in predicting overall survival of patients with icteric leptospirosis

The prognostic value of miR-601 and miR-630 expression to predict mortality was evaluated next. As shown in Fig 3D, serum miR-601 and miR-630 levels could discriminate between survivors and non-survivors with an AUROC of 0.79 (95%CI;0.61–0.98, *P* = 0.016) and 0.82 (95%CI;0.69–0.96, *P* = 0.008), respectively. Based on the ROC, the optimal cut‐off points for miR-601 and miR-630 in predicting mortality was 235.8 and 7.9, respectively.

**Fig 4:**
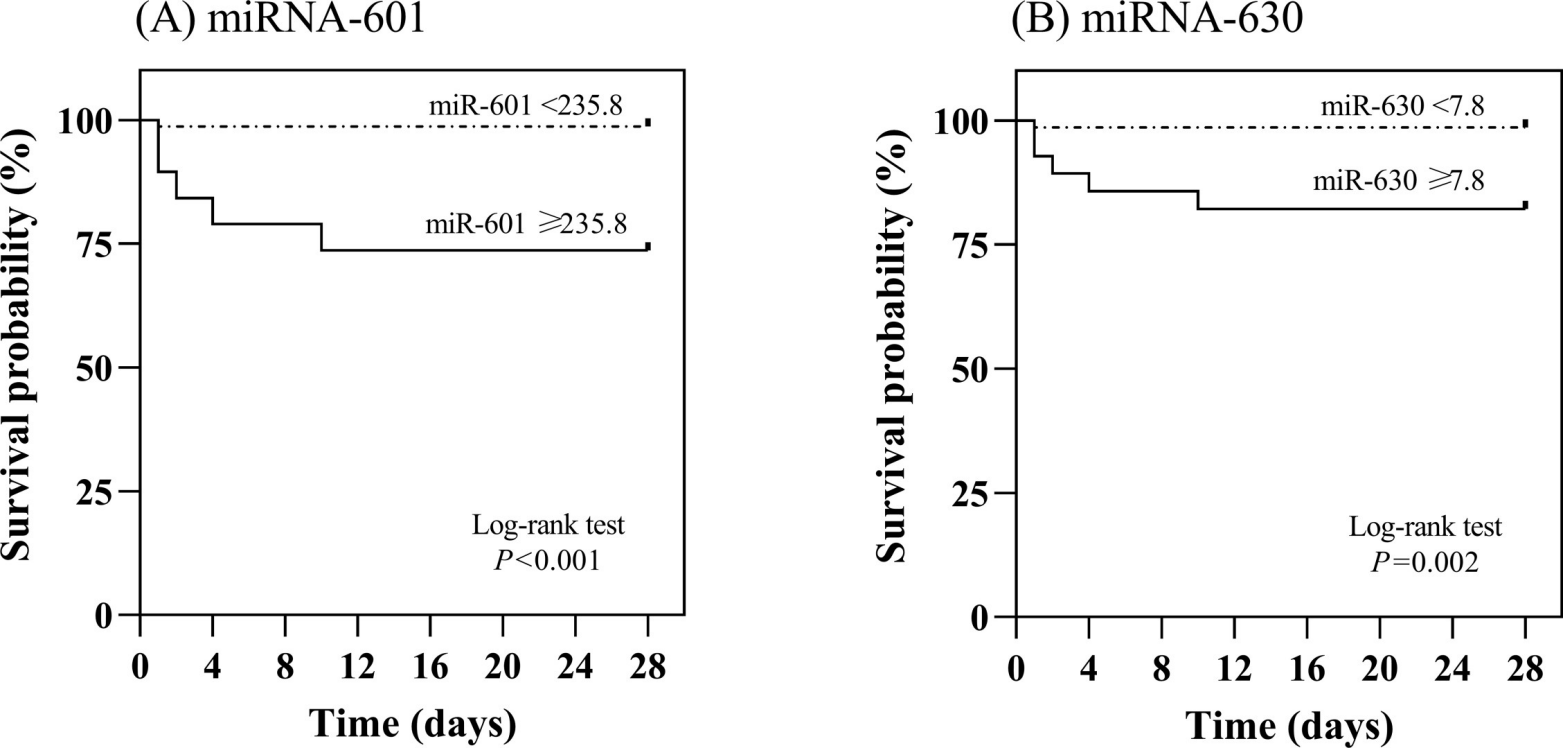
Overall survival analysis of miR-601 (A) and miR-630 (B) in patients with leptospirosis.

We further examined the potential prognostic role of serum miR-601 and miR-630. Using the cut‐off point from the ROC curve, the overall survival of patients with serum miR-601 levels of <235.8 was 27 days, which was significantly better than that of patients whose serum levels were ≥235.8 (overall survival; 21 days, *P*<0.001 by log-rank test) (Fig 4A). Likewise, using the cut‐off points from the ROC curve, the mean overall survival of patients with serum miR-630 levels of <7.9 was 27 days, which was significantly better than that of patients whose serum levels were ≥7.9 (overall survival; 23 days, *P* = 0.002 by log-rank test) (Fig 4B).

### Gene ontology analysis (GO) and KEGG pathway analysis of predicted targets of miRNAs

**Fig 5:**
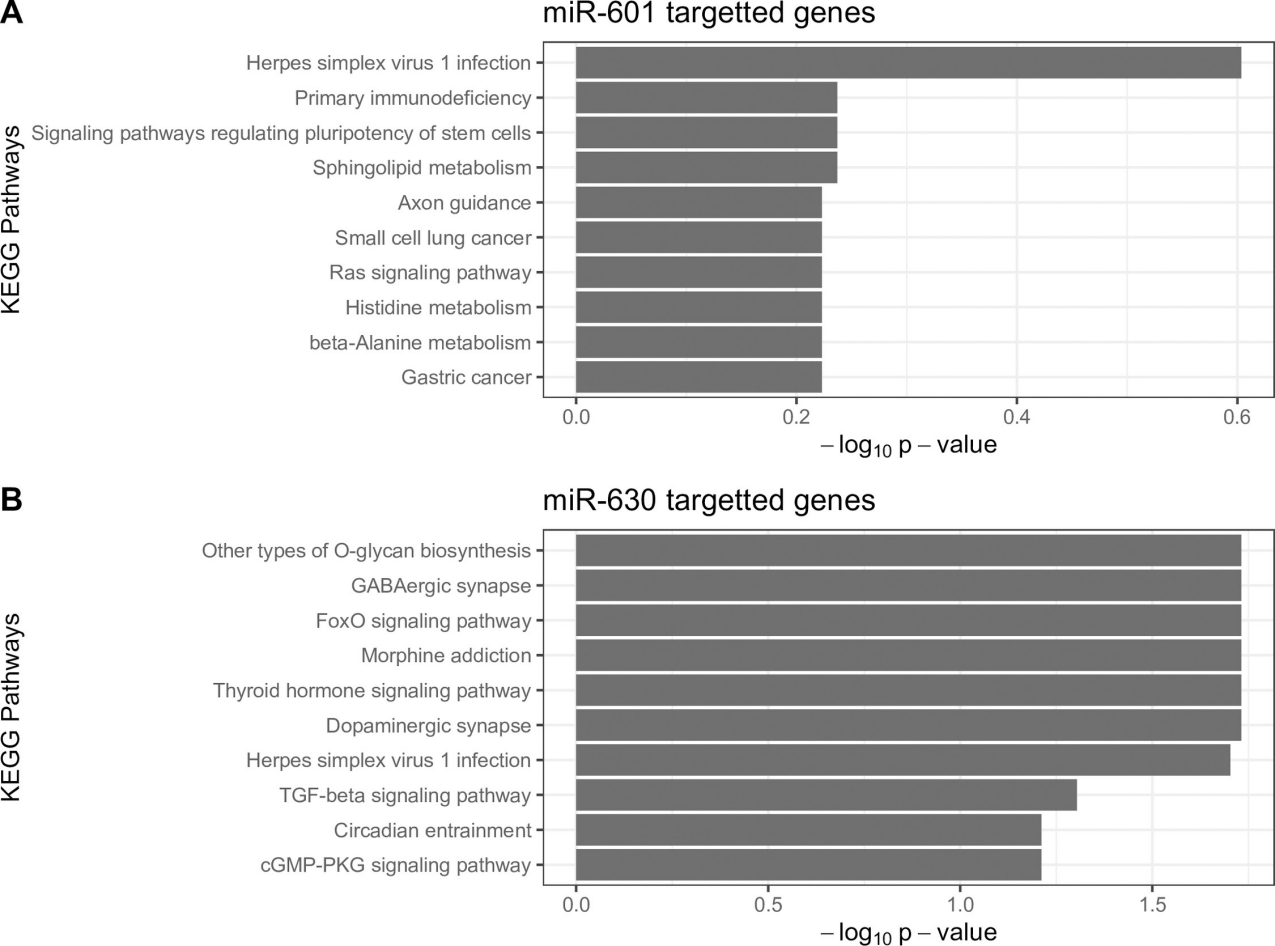
KEGG pathways mapped based on miR-601 (A) and miR-630 (B) candidate target genes.

The prediction of target genes to the differentially expressed miRNAs in patients with icteric leptospirosis was performed by TargetScan program. A total of 1,649 and 1,858 target genes were predicted for miR-601 and miR-630, respectively. All targeted genes were then subjected to GO analysis in clusterProfiler R package. The results demonstrated that these genes were categorized as biological process, molecular function and cellular component. Additionally, pathway analysis based on the predicted targets were identified by KEGG pathway analysis. Our result showed that the two miRNAs were enriched in transforming growth factor (TGF)-β signalling pathway, Ras signalling pathway, pathways in cancers and cell cycle pathways (Fig 5A and 5B).

### Protein‑protein interaction (PPI) and the regulatory network of miRNA–mRNA pathway

**Fig 6:**
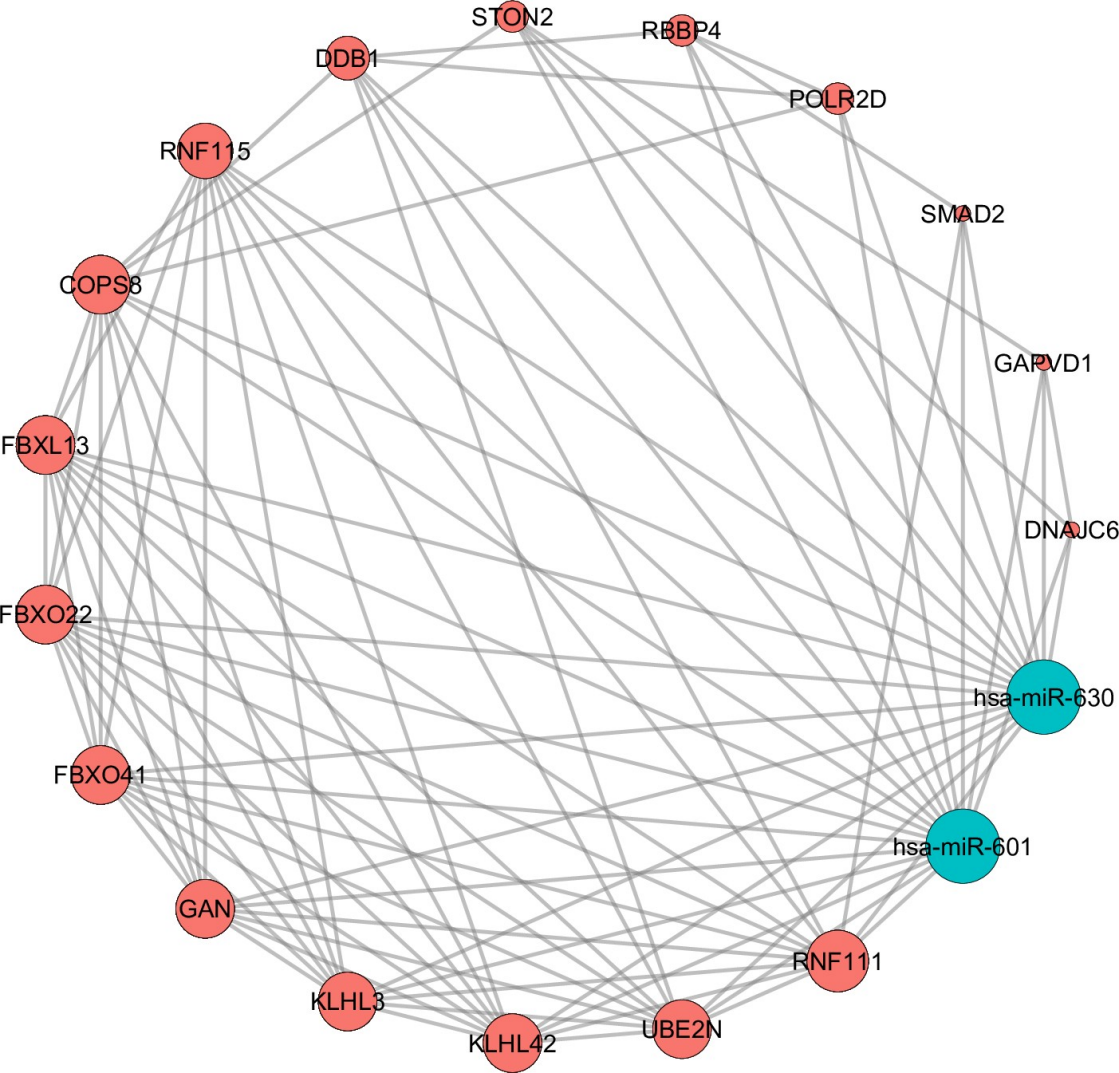
Candidate microRNA (miRNA)-mRNA target network constructed using Cytoscape. 17 genes (Nodes degrees>6.0) and two miRNAs (miR-601 and miR-630) in module.

The gene network analysis of two candidate miR-601 and miR-630 were constructed by STRING software with a high confidence score (combined score of > 0.9) and *P-*value <0.001. A total of 118 nodes with 185 edges were identified in the network analysis. The highest degree of 10 was identified for RNF111 (S3 Fig). To summarize the interaction of these miRNAs and their targeted genes, the miRNA-mRNA network with the nodes degrees >6.0 was contructed using Cytoscape software. Out of 118 nodes, 17 nodes (RNF11, UBE2N, KLHL42, KLHL3, GAN, FBXO41, FBXO22, FBXL13, COPS8, RNF115, DDB1, STON2, RBBP4, POLR2D, GAPVD1 and DNAJC6) showed a degree range from 6 to 10 (Fig 6). These data suggested that these genes might play an important role in the severity of disease and could be used as a potential prognostic biomarkers for patients with icteric leptospirosis.

## Discussion

Leptospirosis represents a neglected disease of global health importance, particularly in the tropics, due to its potentially severe complications with multiple organ involvements [1, 3]. Indeed, the liver is a major target in leptospirosis characterized by a range of clinical manifestations including asymptomatic cases with slight increased in serum transaminases to acute liver failure. In a recent systematic review, it has been shown that markedly increased mortality rates of untreated patients with leptospirosis was positively correlated with the presence of jaundice [5]. For instance, the mortality rate was high in icteric patients (approximately 20%) but very low in individuals without jaundice (0%). Unfortunately, conventional liver function tests unsatisfactorily assess hepatic damage in many patients and may not adequately indicate whether the infection will progress to severe or life-threatening disease. Thus, more effective biomarkers are needed for early prognostic prediction of severe complications.

Considering miRNAs regulate a wide range of cellular processes in immnue response, cumulative evidence has revealed an essential role of these small non-coding RNAs in the diagnosis and prognosis of several infectious diseases [8]. Regarding leptospirosis, *in vitro* data demonstrated that several miRNAs associated with immune regulation were involved in *Leptospira* infection in a virulence- and species-specific manner [20]. Moreover, a previous study identified specific miRNAs that were able to discriminate the infection from other acute febrile diseases with a high sensitivity and specificity [21]. Additionally, our group recently demonstrated distinct expression of circulating microRNAs as novel biomarkers for identifying severe leptospirosis [22]. However, there is very limited information regarding the expression of circulating miRNAs in patients with leptospirosis in the context of liver-related complications.

The present study was aimed to compare miRNAs expression by the NanoString platform in serum samples of patients with anicteric and icteric leptospirosis, and then validated selected candidate miRNAs in a large-scale samples by qRT-PCR. In the discovery cohort, we identified 38 miRNAs differentially expressed between the studied groups. Among these, miR-601 and miR-630 were selected as the top two candidates. In the validated cohort, we confirmed that serum miR-601 and miR-630 levels were significantly higher in patients with the icteric group compared with the anicteric group. These miRNAs concentrations were also correlated with worse clinical parameters such as blood urea nitrogen, creatinine and total bilirubin levels. Based on ROC curve, it was shown that circulating miR-601 and miR-630 could satisfactory discriminate the icteric group from the anicteric group. In addition, high miR-601 or miR-630 expression levels were able to predict patients with a high probability of subsequent developing acute liver failure. Notably, the sensitivity of miR-601 and miR-630 in predicting acute liver failure was 100%. Thus, it appeared from our results that both markers could allow the prediction of leptospirosis-induced acute liver failure with a very high sensitivity. Moreover, our data demonstrated that miR-601 and miR-630 were beneficial for predicting acute liver failure, multi-organ failures and overall survival of patients with icteric leptospirosis.

Currently, little is known about the role of miR-601 and miR-630 in infectious diseases. In previous studies, miR-601 was mainly described to be involved in cancer development and prognosis of several types of cancer, including gastric cancer and non-small-cell lung cancer (NSCLC) [23, 24]. For instance, serum miR-601 was not only identified as a promising molecular signature for early detection but was also associated with adverse clinical outcome and poor survival of patients with NSCLC [24]. Regarding miR-630, this miRNA was also found to be up-regulated in various malignancies such as gastric cancer, NSCLC and head and neck squamous cell carcinoma [25–28]. In hepatocellular carcinoma, it was demonstrated that low miR-630 expression in liver tissue was associated with higher recurrence rates and shorter overall survival in comparison with those with high tissue expression. Moreover, functional analysis indicated that miR-630 exerted tumor-suppressor functions and its overexpression could attenuate the epithelial-mesenchymal transition (EMT) phenotype [29].

To further elucidate the molecular pathogenesis of icteric leptospirosis, *in-silico* analysis for miR-601 and miR-630 was performed. In this context, the enriched KEGG pathways were found to be involved in various biological processes and immunity, indicating their potential roles in modulating immune responses and disease pathogenesis. For instance, TGF-β, a potent multi-functional cytokine, plays essential roles in immunomodulatory effects under pathophysiological conditions such as autoimmunity and infection [30]. In animal models of leptospirosis, the triggering of inflammatory response, particularly through the excessive production of pro-inflammatory cytokines including interleukins (IL)-1β, IL-6, IL-12 and tumor necrosis factors (TNFs) could lead to activating immunomodulatory cytokines such as IL-4, IL-10, IL-13 and TGF-β, which result in a sepsis-like syndrome associated with organ failures [31]. Regarding Ras signalling pathway, while currently highlighting within the field of cancer biology, this superfamily of small GTPases also plays fundamental roles in immunity and inflammation [32]. Moreover, the expression of main potential innate immunity and inflammation signaling molecules such as RNF11, UBE2N and RNF115 [33–35] were found to have high degree of interaction in the PPI network.

The strength of our study was the well-characterized prospective observational cohort using the stored samples of patients with confirmed leptospirosis by standard methods. Additionally, the serum samples were collected on the first day of clinical presentation, which could be more feasible than the collection at the first day of symptoms. However, some limitations should be noted. First, in this study there was a relatively small number of patients with icteric leptospirosis that could decrease the power of statistical analysis. However, the number of icteric cases in our report represented a real-world cohort, which was in line with the median value across patient series (approximately 20%) in the recent systematic review [5].

Additionally, selecting candidate miRNAs for validation by qRT-PCR was based on their consistently up- or down-regulated expressions and their relevance to immune response and inflammation. Thus, miR-548n that was down-regulated in RNA-seq analysis was not selected for further analysis due to its unknown pathobiology. Finally, we evaluated serum biomarkers at the initial visit, but did not assess their sequential changes during hospitalization or follow-up period.

To summarize our study, the NanoString assay revealed several miRNAs differentially expressed in serum samples of patients with icteric leptospirosis as compared to the unicteric group. Among these miRNAs, circulating miR-601 and miR-630 were up-regulated in patients with icteric leptospirosis and displayed good predictors of subsequent acute liver failure. In addition, these miRNAs were associated with multi-organ failures and overall survival of patients. However, further investigations are required to confirm our observations and to elucidate the biological functions of these miRNAs contribute to disease severity in patients with leptospirosis.

## Supporting information

S1 Fig Heatmap of 38 miRNAs expression between patients with anicteric and icteric leptospirosis. (TIF)

S2 Fig ROC curves of combined miR-601 and miR-630 levels. (TIF)

S3 Fig miRNA-mRNA network; a total of 118 nodes with 185 edges. (PDF)

S1 Table The differentially expressed miRNAs between patients with anicteric and icteric leptospirosis. (XLSX)
