# Supplementary figures and images for: Identification and validation of circulating miRNAs as potential new biomarkers for severe liver disease in patients with leptospirosis
Source: PLoS One. 2021 Sep 27;16(9):e0257805. doi: 10.1371/journal.pone.0257805 (PMC8476044; doi:10.1371/journal.pone.0257805)

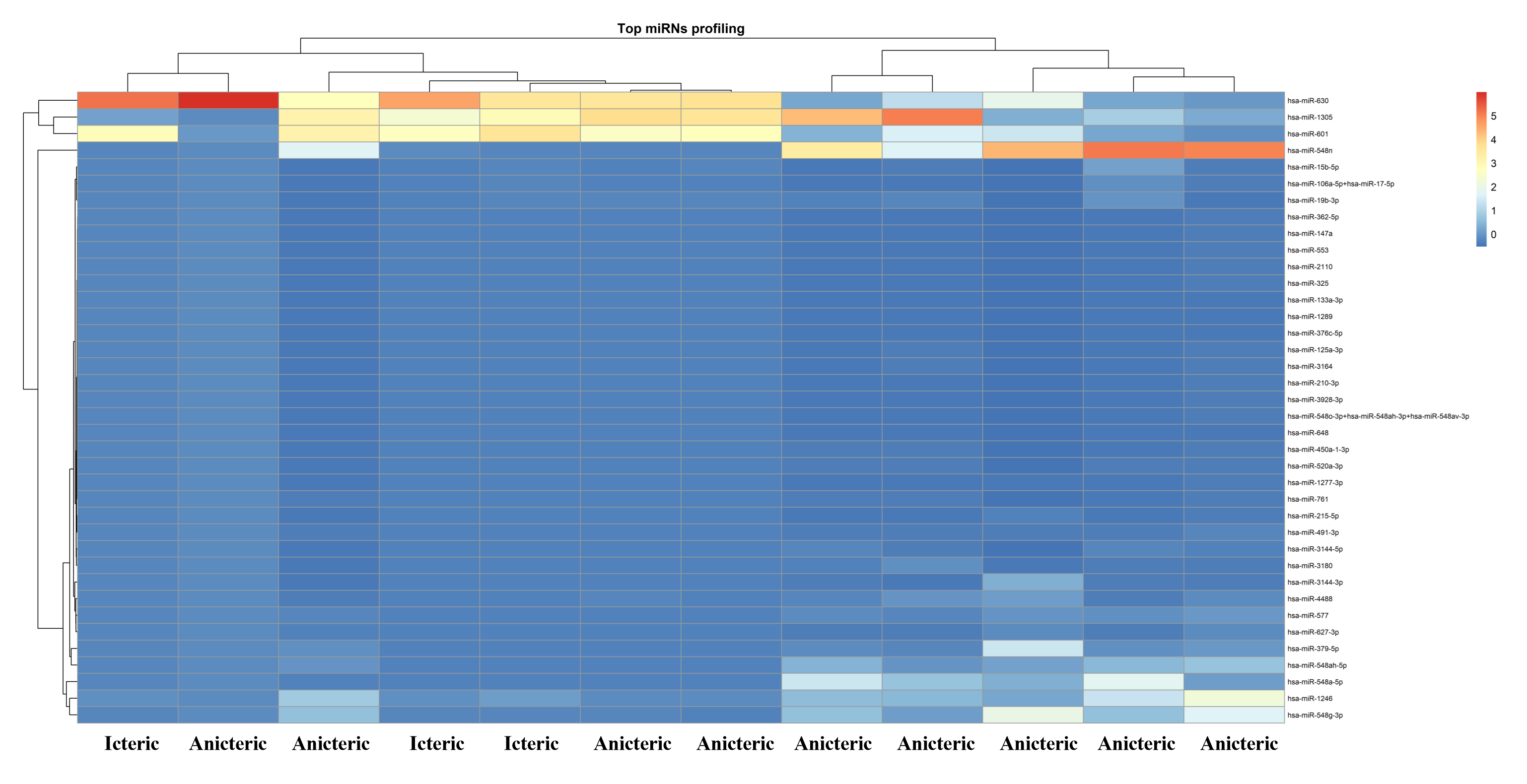

Supplement: S1 Fig — (TIF) [file pone.0257805.s001.tif]

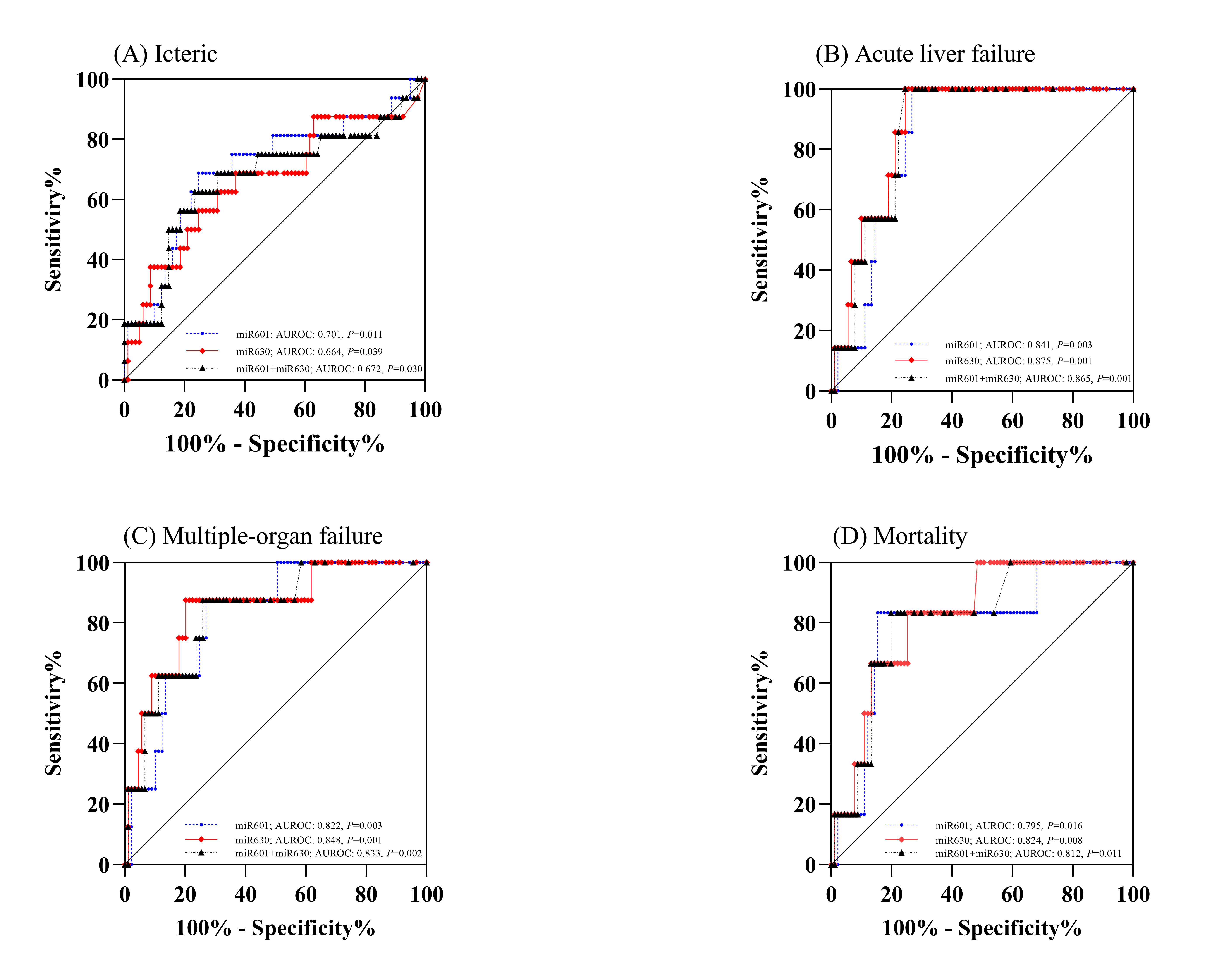

Supplement: S2 Fig — (TIF) [file pone.0257805.s002.tif]

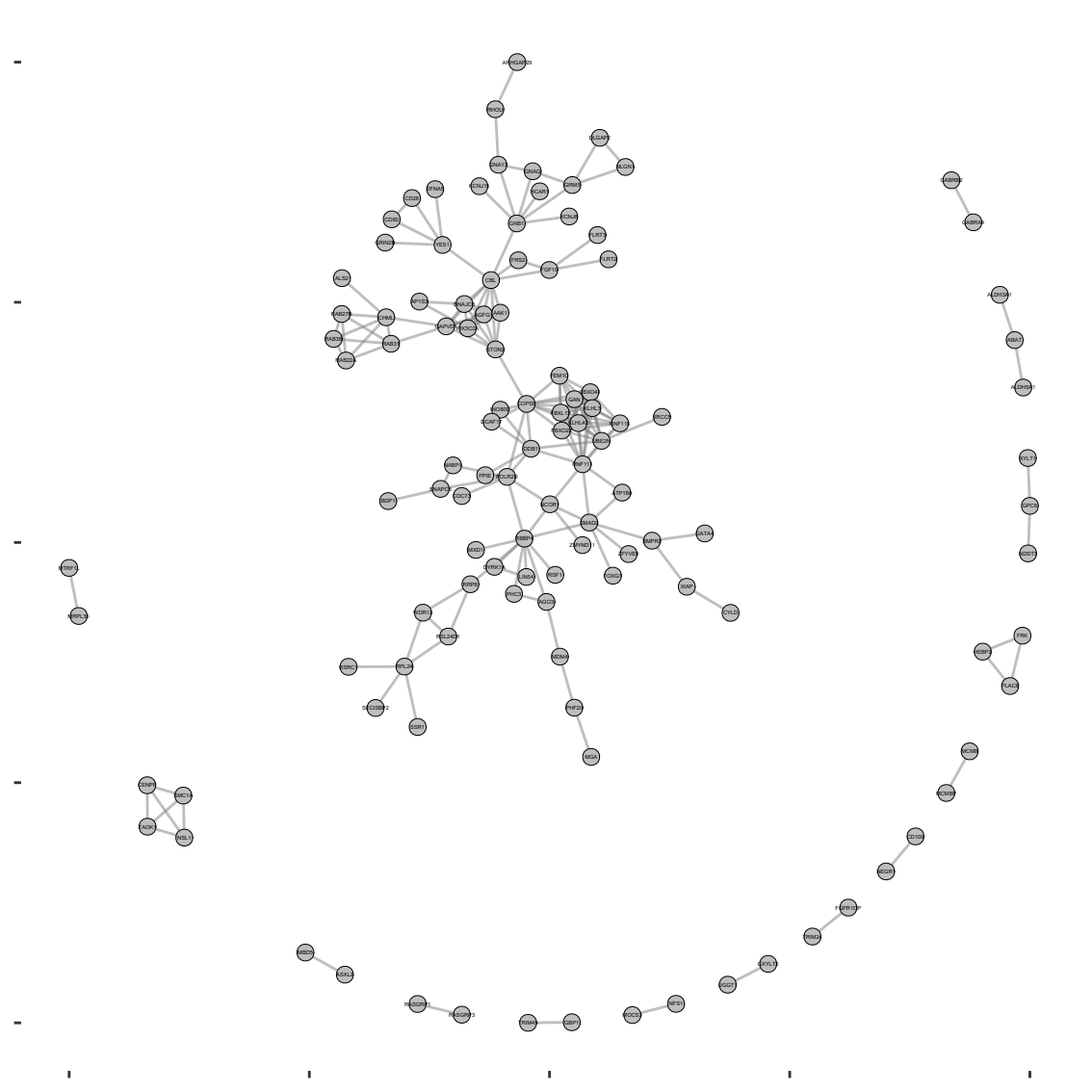

Supplement: S3 Fig — (PDF) [file pone.0257805.s003.pdf]
